# Anamorphic development and extended parental care in a 520 million-year-old stem-group euarthropod from China

**DOI:** 10.1186/s12862-018-1262-6

**Published:** 2018-09-29

**Authors:** Dongjing Fu, Javier Ortega-Hernández, Allison C Daley, Xingliang Zhang, Degan Shu

**Affiliations:** 1 Department of Geology, State Key Laboratory of Continental Dynamics, Shaanxi Key Laboratory of Early Life and Environment, Northwest University, Xian, 710069 People’s Republic of China; 2 Department of Zoology, University of Cambridge, Downing Street, Cambridge, CB2 3EJ UK; 3 Museum of Comparative Zoology and Department of Organismic and Evolutionary Biology, Harvard University, 26 Oxford Street, Cambridge, MA 02138 USA; 4 Institute of Earth Sciences, University of Lausanne, Géopolis, CH-1015 Lausanne, Switzerland

**Keywords:** *Fuxianhuia*, Chengjiang, Post-embryonic development, Hemianamorphosis, Heterochrony, Reproductive ecology

## Abstract

**Background:**

Extended parental care is a complex reproductive strategy in which progenitors actively look after their offspring up to – or beyond – the first juvenile stage in order to maximize their fitness. Although the euarthropod fossil record has produced several examples of brood-care, the appearance of extended parental care within this phylum remains poorly constrained given the scarcity of developmental data for Palaeozoic stem-group representatives that would link juvenile and adult forms in an ontogenetic sequence.

**Results:**

Here, we describe the post-embryonic growth of *Fuxianhuia protensa* from the early Cambrian Chengjiang Lagerstätte in South China. Our data demonstrate anamorphic post-embryonic development for *F. protensa*, in which new tergites were sequentially added from a posterior growth zone, the number of tergites varies from eight to 30. The growth of *F. protensa* is typified by the alternation between segment addition, followed by the depletion of the anteriormost abdominal segment into the thoracic region. The transformation of abdominal into thoracic tergite is demarcated by the development of laterally tergopleurae, and biramous walking legs. The new ontogeny data leads to the recognition of the rare Chengjiang euarthropod *Pisinnocaris subconigera* as a junior synonym of *Fuxianhuia.* Comparisons between different species of *Fuxianhuia* and with other genera within Fuxianhuiida suggest that heterochrony played a prominent role in the morphological diversification of fuxianhuiids. Functional analogy with the flexible trunk ontogeny of Cambrian and Silurian olenimorphic trilobites suggests an adaptation to sporadic low oxygen conditions in Chengjiang deposits for *F. protensa*. Finally, understanding the growth of *F. protensa* allows for the interpretation of an exceptional life assemblage consisting of a sexually mature adult alongside four ontogenetically coeval juveniles, which constitutes the oldest occurrence of extended parental care by prolonged cohabitation in the panarthropod fossil record.

**Conclusions:**

Our findings constitute the most detailed characterization of the post-embryonic development in a soft-bodied upper stem-group euarthropod available to date. The new ontogeny data illuminates the systematics, trunk segmentation and palaeoecology of *F. protensa*, offers insights on the macroevolutionary processes involved in the diversification of this clade, and contributes towards an improved understanding of complex post-embryonic reproductive ecology in Cambrian euarthropods.

**Electronic supplementary material:**

The online version of this article (10.1186/s12862-018-1262-6) contains supplementary material, which is available to authorized users.

## Background

Fuxianhuiida represents a distinctive clade of upper stem-group euarthropods exclusively known from the early Cambrian of South China [1–4], and figure among the most thoroughly scrutinized Lower Palaeozoic taxa owing to several remarkable instances of soft-tissue preservation [5–11]. Despite their contribution towards understanding the origin of Euarthropoda [1–11], basic aspects of the post-embryonic development of fuxianhuiids and most other stem lineage representatives remain largely uncharted [12]. Although it has been recognized that fuxianhuiid populations include individuals of different sizes and variable number of exoskeletal trunk tergites suggesting the occurrence of different ontogenetic stages [1–3, 5], there is no formal description of this variability nor its implications for the palaeobiology of these animals. Some studies have produced remarkable insights on the post-embryonic development in other Cambrian stem-group euarthropods, such as the recognition of limb rudiments in a larva of the megacheiran *Leanchoilia illecebrosa* [13], and the changes in bivalved carapace morphology and body segment count during growth in *Isoxys auritus* [14]. However, detailed information on the ontogeny and reproduction of Lower Palaeozoic euarthropods is for the most part only available from crown-group members [15–25], precluding the ancestral reconstruction of these traits during the early evolution of the phylum. Here we describe the post-embryonic development of *Fuxianhuia protensa* from the Cambrian (Stage 3) Chengjiang biota in South China [1, 5]. This represents the most comprehensive characterization of the ontogeny in a stem-group euarthropod to date, and leads to the recognition of parental care in fuxianhuiids, casting new light on the complex reproductive behaviour of early animals during the Cambrian Explosion.

## Results

**Fig. 1:**
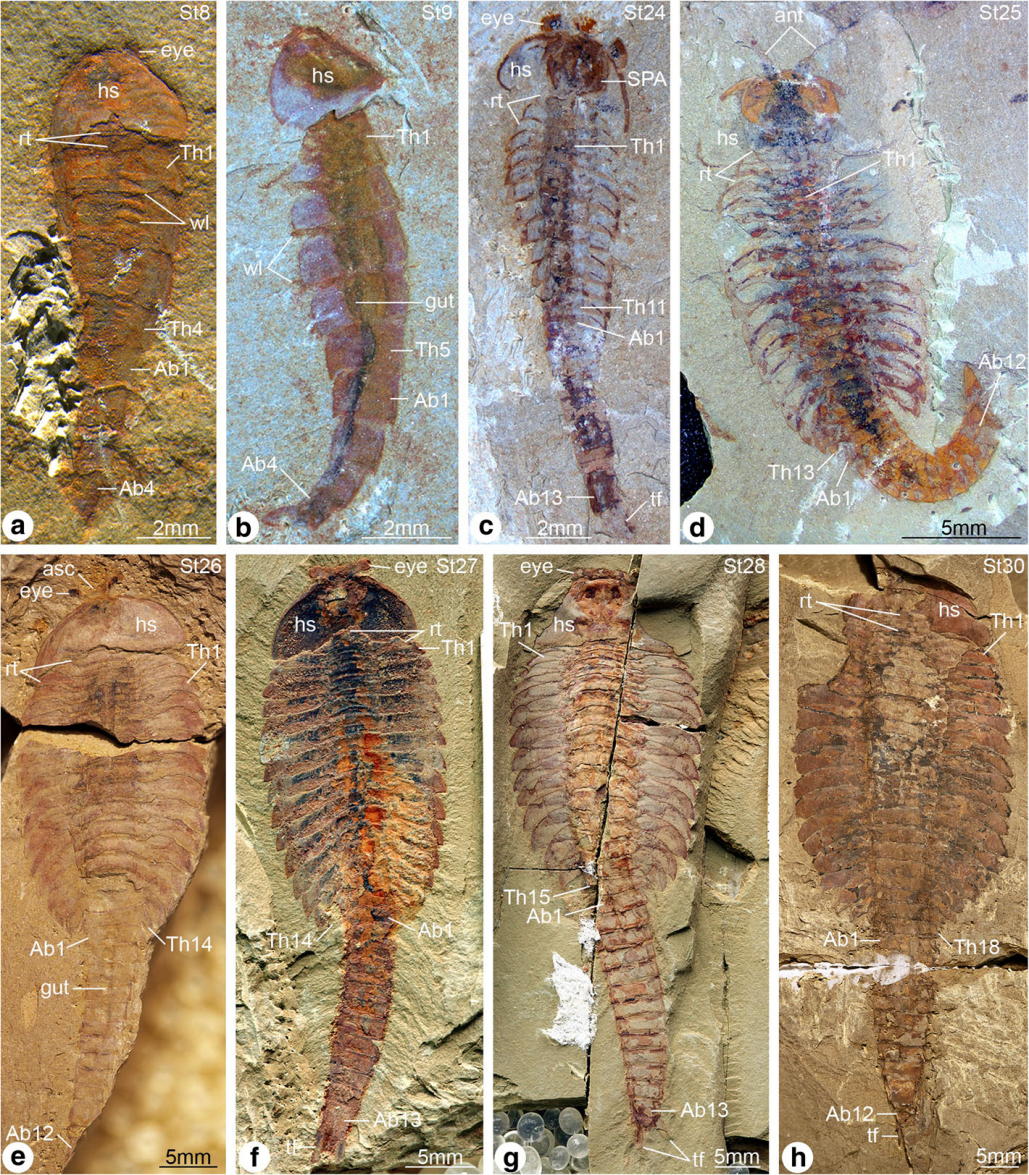
Ontogenetic stages of *Fuxianhuia protensa* from the early Cambrian Chengjiang Lagerstätte in South China. **a** ELI 0722A, stage 8; **b**) ELI 0728, stage 9; (**c**) ELI 0034, stage 24a; (**d**) ELI 0050, stage 25b; (**e**) ELI 0001A, stage 26b; (**f**) ELI 36–77, stage 27a; (**g**) ELI 520-27A, stage 28a; (**h**) ELI 0011, stage 30b. Ab*n*, abdominal tergite; ant, antennae; asc, anterior sclerite; hs, head shield; rt., reduced anterior tergites; tf, tail flukes; Th*n,* thoracic tergite; *wl*, walking legs

**Fig. 2:**
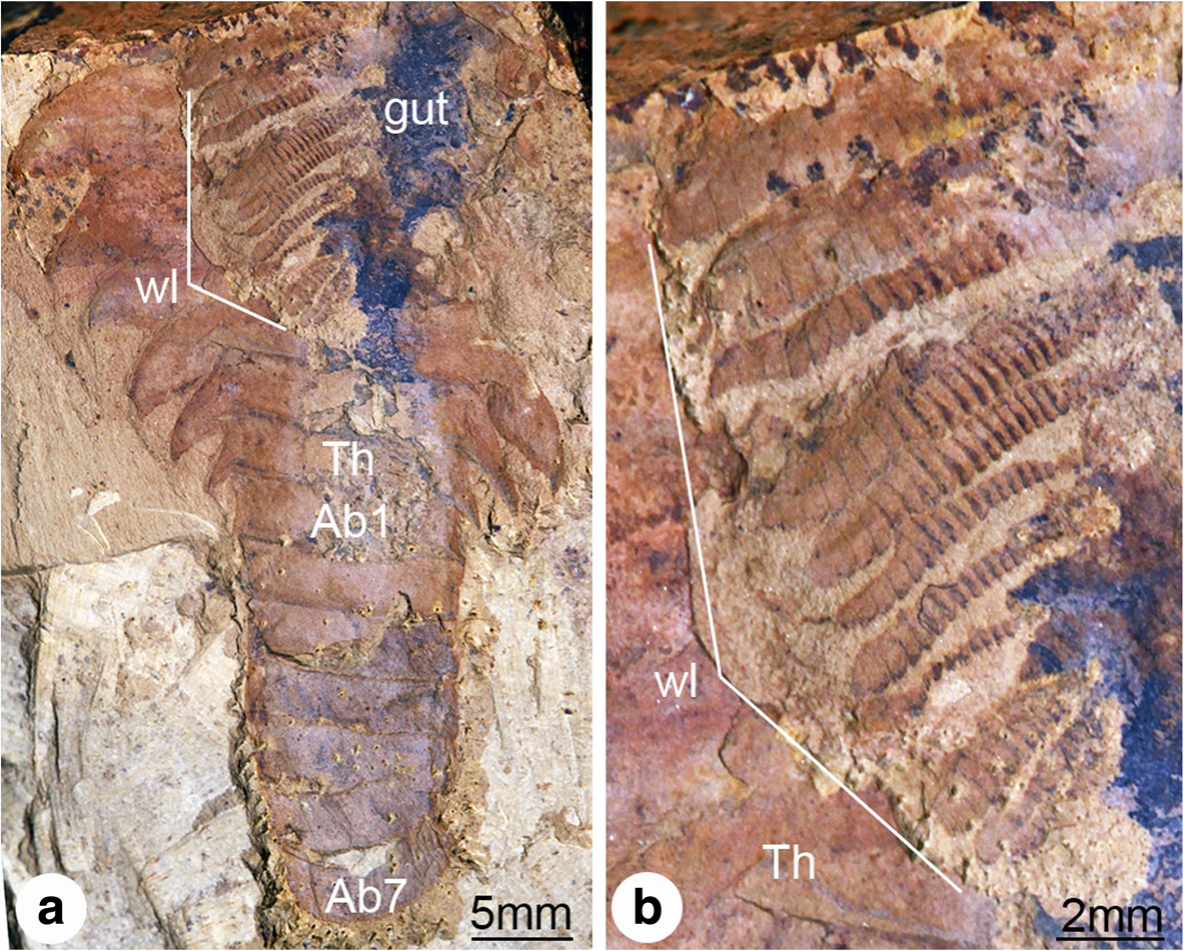
Trunk limb development in *Fuxianhuia protensa* from the early Cambrian Chengjiang Lagerstätte in South China. **a** CJ1069, articulated adult specimen of uncertain ontogenetic stage with dorsal exoskeleton prepared to illustrate the presence of multiple pairs of trunk appendages associated with each trunk tergite; note the morphological distinction between the limb-bearing thorax with expanded tergopleurae and the limb-less narrow abdomen. **b** Magnification of trunk appendages; note that endopods are less developed, smaller in size and have less podomeres towards the posterior end of the thorax. Ab*n*, abdominal tergite; Th*n,* thoracic tergite; *wl,* walking legs

**Fig. 3:**
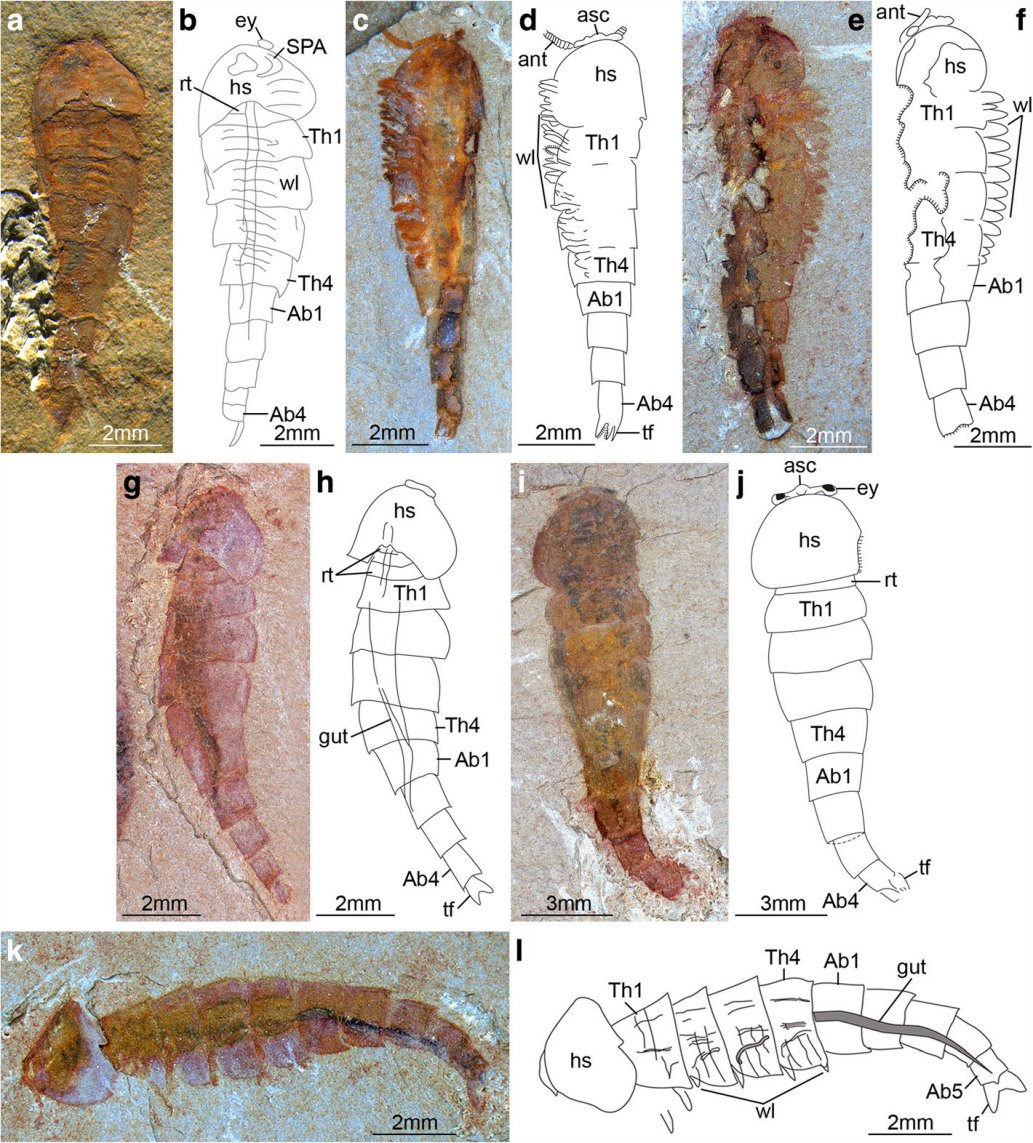
*Fuxianhuia protensa* stages 8 and 9. **a**-**j** Stage 8; (**a** and **b**) ELI 0722A; (**c** and **d**) ELI 0723A; (**e** and **f**) ELI 0731; (**g** and **h**) ELI MU76A-c; (**I** and **j**) ELI MU76B-b; (**k** and **l**) ELI 0728, stage 9. Ab*n*, abdominal tergite; ant, antennae; asc, anterior sclerite; hs, head shield; rt., reduced anterior tergites; SPA, specialized post-antennal appendage; tf, tail flukes; Th*n,* thoracic tergite; *wl*, walking legs

Complete individuals of *F. protensa* vary in total length from 1 to 8 cm (Fig. 1). All ontogenetic stages share a fundamentally similar body construction. The head comprises a (protocerebral) anterior sclerite with paired stalked eyes [26], a pair of pre-oral (deutocerebral) antennae [8], and a pair of para-oral (tritocerebral) specialized post-antennal appendages [2]. Dorsally, the anterior sclerite articulates with a subtrapezoidal head shield, whose proportions range from a 1:1 length/width ratio in juveniles (Fig. 1a*,* b; Fig. 3) to a wider 1:4 length/width ratio in later stages (Fig. 1c*-*h; Figs 5, 6, 7, 8, 9 and 10). The trunk consists of a variable number of overlapping tergites and a terminal tailspine with paired caudal flukes. Although the trunk expresses most of the ontogenetic changes, there are some invariable aspects of its organization. 1) The head shield covers - but is not fused to - three reduced anteriormost tergites, each of which bears a single limb pair but lacks laterally expanded tergopleurae [1, 5]. 2) Trunk tergites gradually change in size posteriorly. 3) The trunk is subdivided into an anterior limb-bearing thorax with expanded tergopleurae, and a posterior limb-less abdomen composed of narrow cylindrical tergites. 4) Thoracic tergites are associated with two or three pairs of biramous walking legs as a result of a derived pattern of ventral segmental mismatch [1, 5, 27]. 5) The trunk endopods are largest in size and possess more podomeres towards the anterior half of the body across all ontogenetic stages, whereas they have a reduced size and number of podomeres towards the posterior, with the smallest limbs located underneath the last thoracic tergite [1, 5] (Fig. 2; Fig. 3a*-*f).

**Fig. 4:**
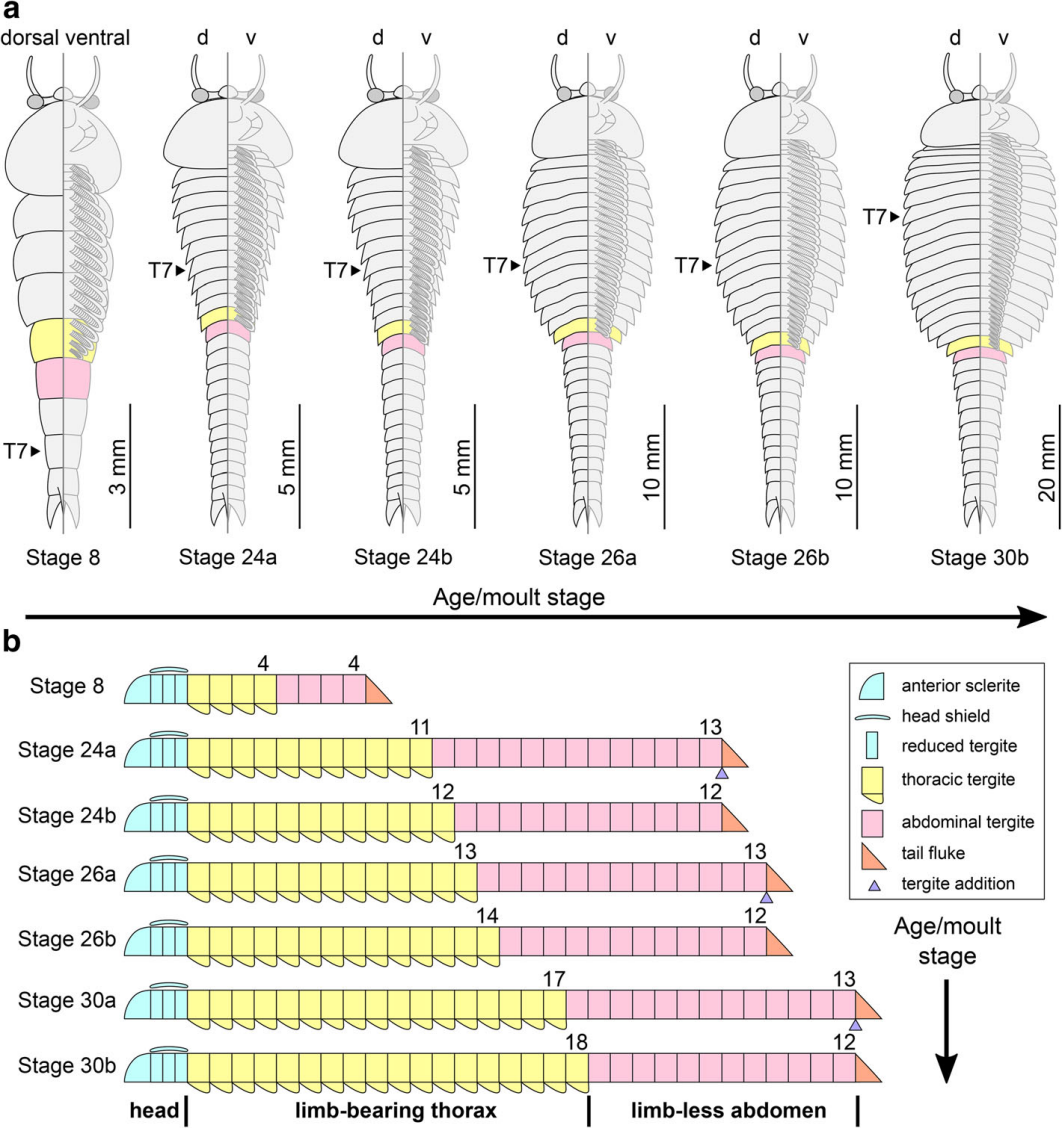
Ontogenetic changes in trunk region of *Fuxianhuia protensa* during post-embryonic development. **a** Stage 8 is the earliest juvenile available, and possesses 4 thoracic limb-bearing tergites with short tergopleurae, followed by 4 limb-less cylindrical tergites. The three reduced anteriormost tergites under the head shield remain invariant. Throughout ontogenetic development, the most anterior abdominal tergite (pink) develops expanded pleurae and walking legs, transforming into the most posterior thoracic tergite (yellow). Tergite 7 arrowed in all stages for comparison. Stage 30b is the oldest known phase. **b** Partial trunk segmentation schedule for *F. protensa*; complete ontogenetic reconstruction provided in Fig. 11

**Fig. 5:**
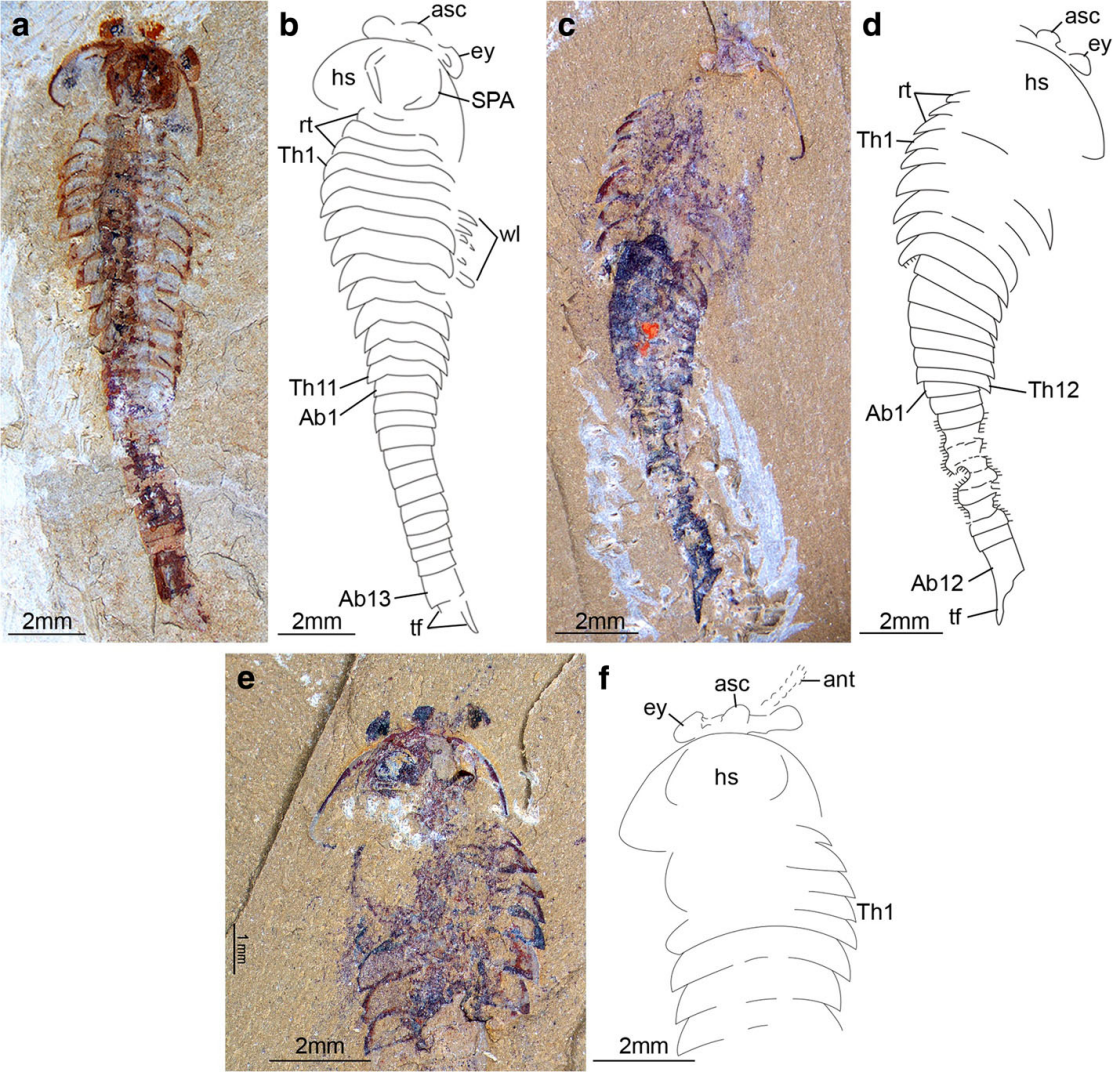
*Fuxianhuia protensa* stage 24. **a** and **b** ELI 0034, stage 24a; (**c** and **d**) ELI 0373A, stage 24b; (**e** and **f**) ELI 0373B; counterpart, stage 24b. Ab*n*, abdominal tergite; ant, antennae; asc, anterior sclerite; hs, head shield; rt., reduced anterior tergites; SPA, specialized post-antennal appendage; tf, tail flukes; Th*n,* thoracic tergite; *wl*, walking legs

**Fig. 6:**
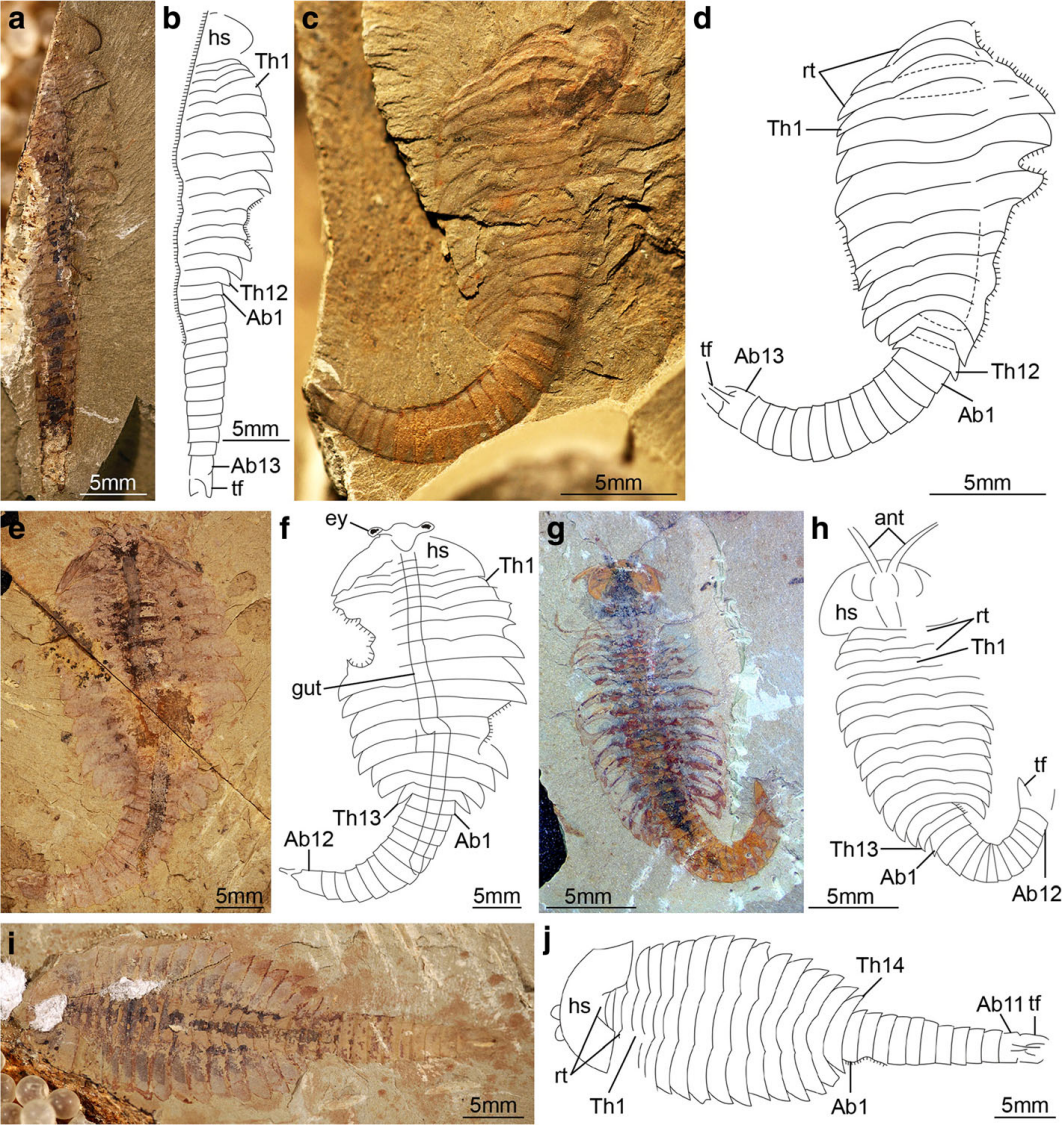
*Fuxianhuia protensa* stage 25. **a**-**d** Stage 25a; (**a** and **b**) ELI 0469; (**c** and **d**) ELI 0591; (**e**-**h**) Stage 25b; (**e** and **f**) ELI 0076; (**g** and **h**) ELI 0050; (**i** and **j**) ELI 0276, stage 25 individual with 14 thoracic tergites and 11 abdominal tergites. Ab*n*, abdominal tergite; ant, antennae; hs, head shield; rt., reduced anterior tergites; tf, tail flukes; Th*n,* thoracic tergite

**Fig. 7:**
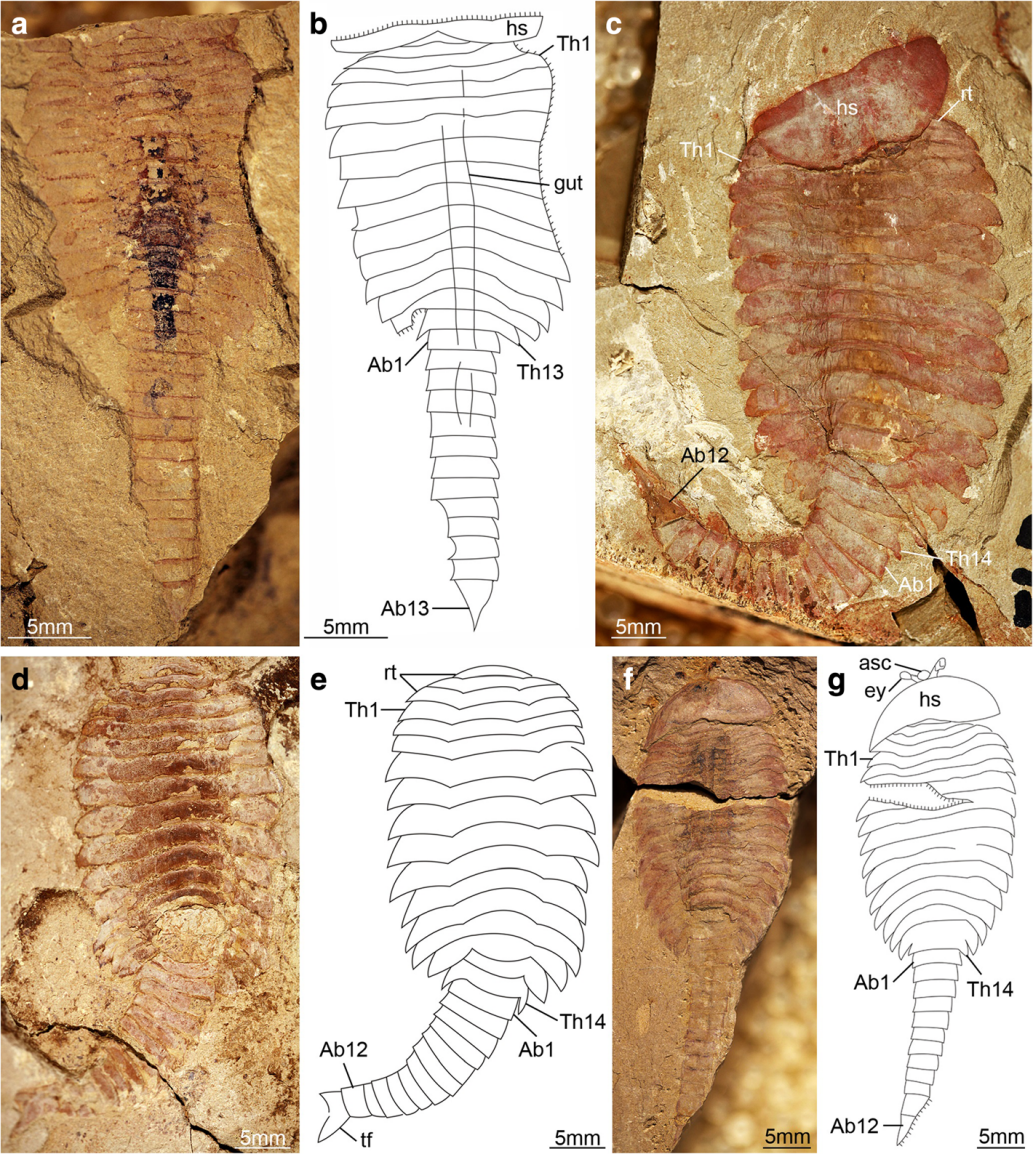
*Fuxianhuia protensa* stage 26. **a** and **b** ELI 0073, Stage 26a; (**c**-**g**) Stage 26b; (**c**) ELI 0006A; (**d** and **e**) ELI 0052B; (**f** and **g**) ELI 0001A. Ab*n*, abdominal tergite; asc, anterior sclerite; hs, head shield; rt., reduced anterior tergites; tf, tail flukes; Th*n,* thoracic tergite

**Fig. 8:**
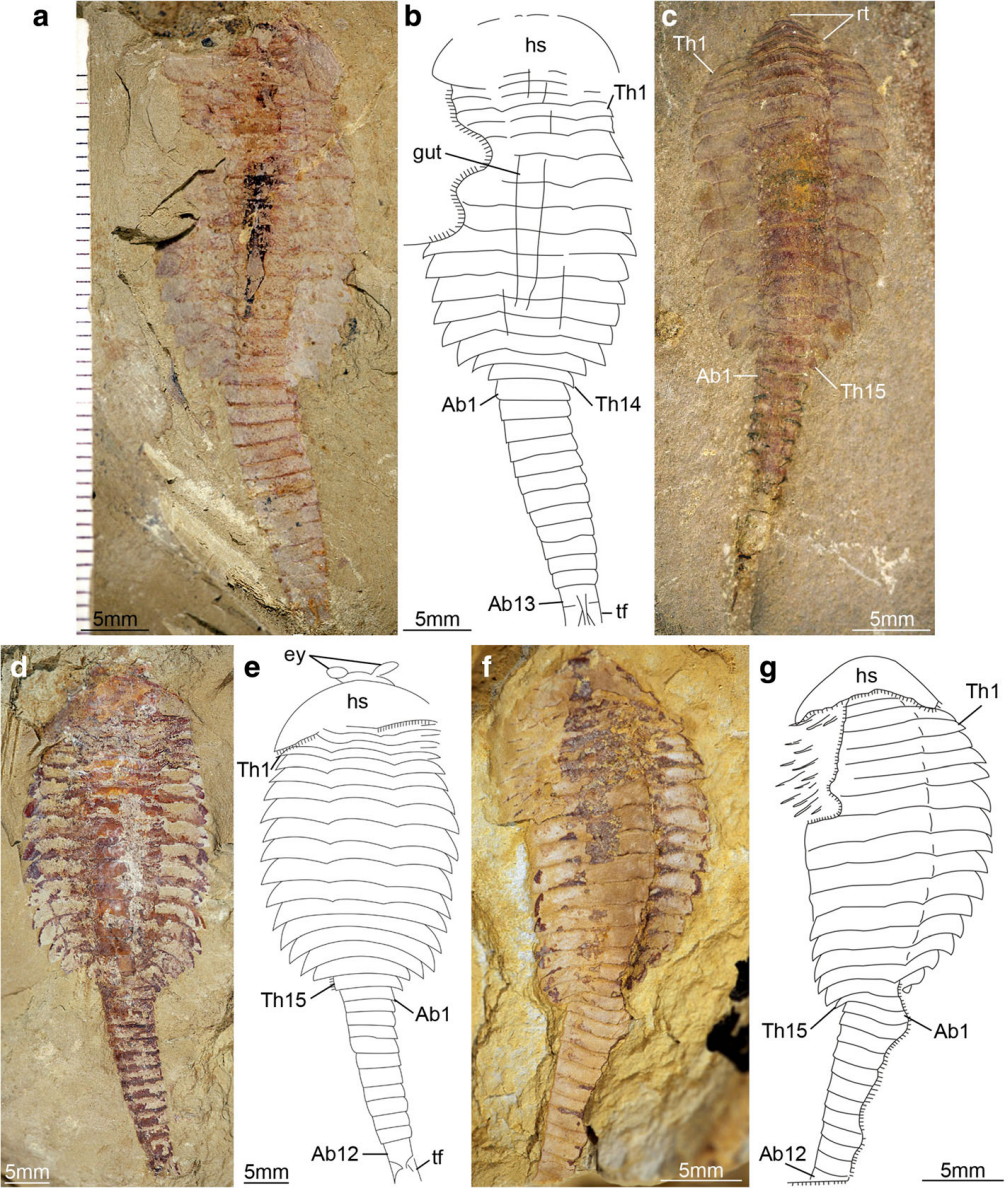
*Fuxianhuia protensa* stage 27. **a** and **b** ELI 0073A, stage 27a; (**b**-**g**) Stage 27b; (**c**) ELI 0131A; (**d** and **e**) ELI 0092A; (**f** and **g**) ELI 0123B. Ab*n*, abdominal tergite; hs, head shield; rt., reduced anterior tergites; tf, tail flukes; Th*n,* thoracic tergite

**Fig. 9:**
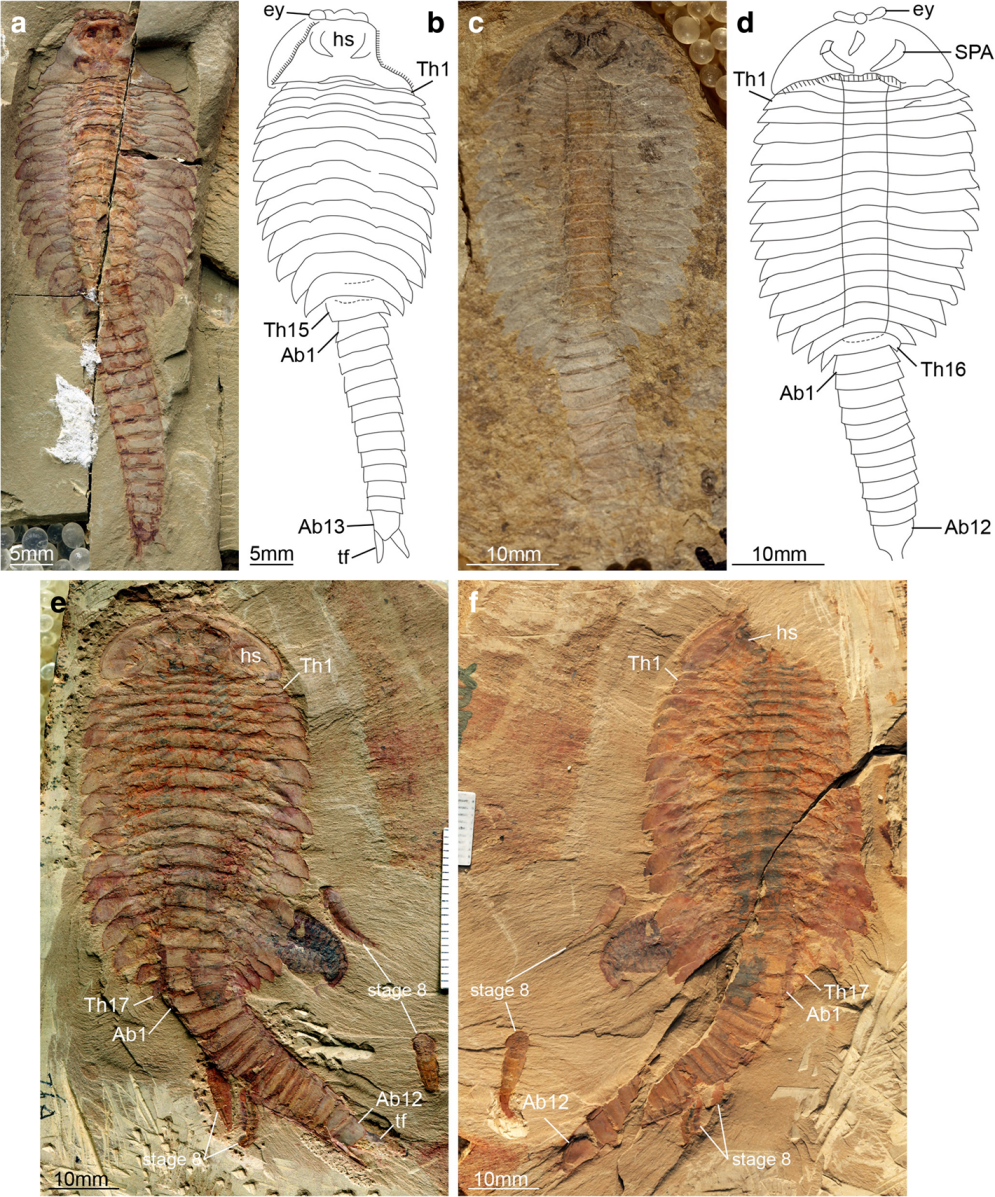
*Fuxianhuia protensa* stage 28 and 29. **a** and **b** ELI 520-27A, Stage 28a; (**c** and **d**) ELI 0641, stage 28b; (**e**) ELI MU76A, part, stage 29; (**f**) ELI MU76B, counterpart, Stage 29. Ab*n*, abdominal tergite; hs, head shield; rt., reduced anterior tergites; tf, tail flukes; Th*n,* thoracic tergite

**Fig. 10:**
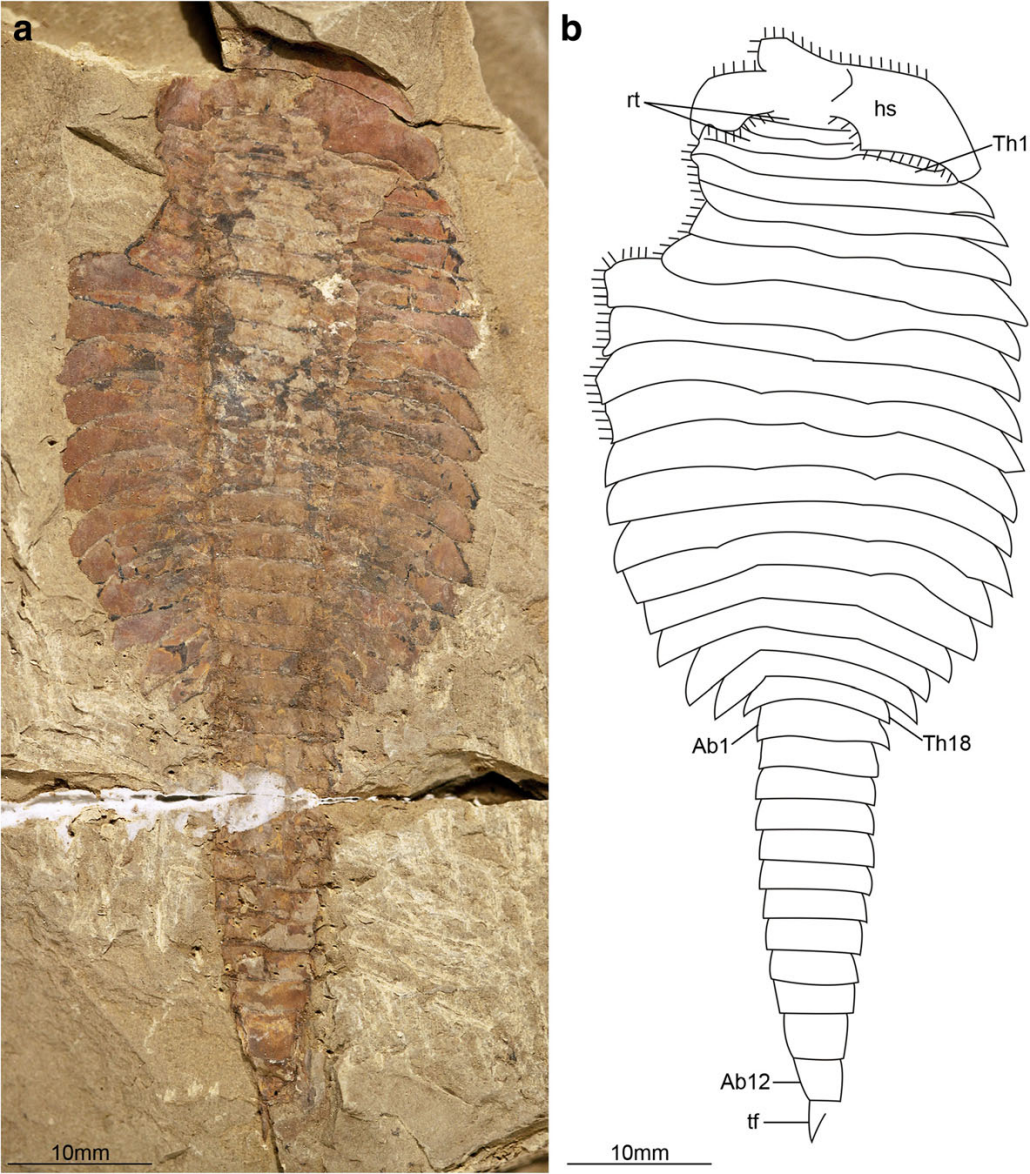
*Fuxianhuia protensa* stage 30b. **a** and **b** ELI 0011. Ab*n*, abdominal tergite; hs, head shield; rt., reduced anterior tergites; tf, tail flukes; Th*n,* thoracic tergite

**Fig. 11:**
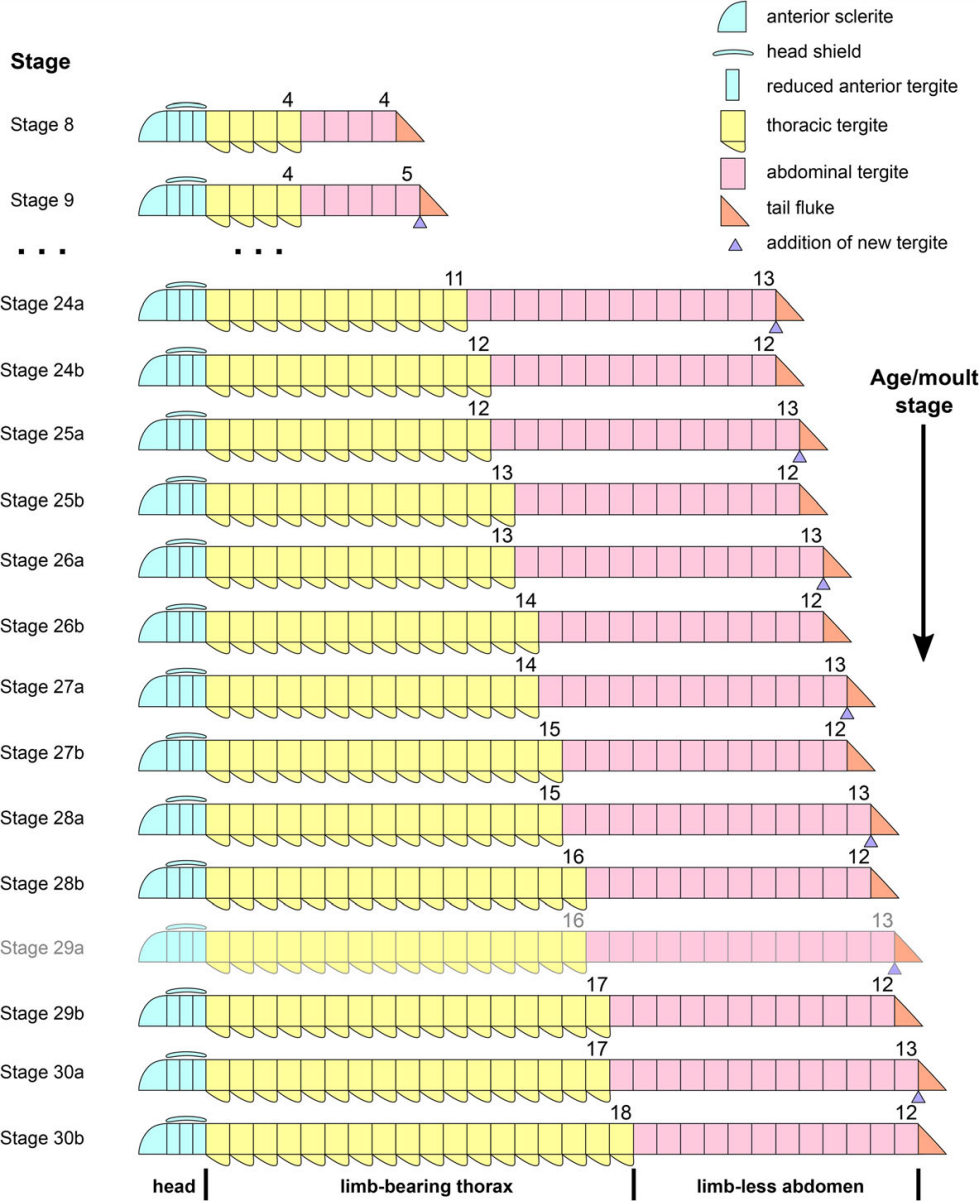
Reconstruction of the trunk segmentation schedule of *Fuxianhuia protensa*. Note that stage 29a specimens have not been found

**Fig. 12:**
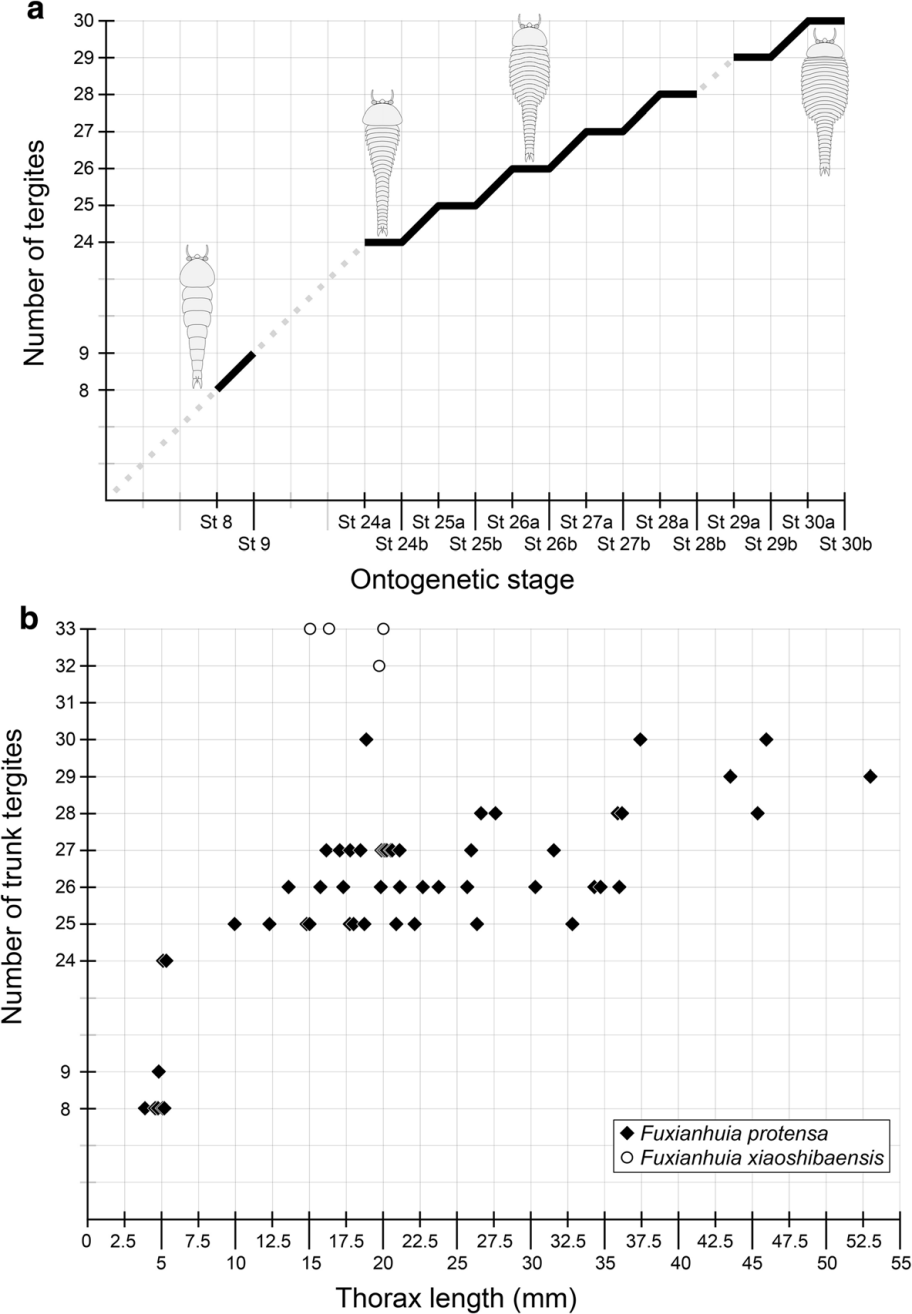
Patterns of ontogenetic development in *Fuxianhuia*. **a** Ontogenetic trajectory of *Fuxianhuia protensa*, illustrating alternation between phases of posterior segment addition (slopes), and abdominal to thoracic tergite transformation (plateaus). Dashed lines denote unavailable ontogenetic stages. **b** Relationship between ontogenetic stage and body size, as expressed by thoracic length, in articulated individuals of *F. protensa* (*n* = 57) (Figs. 1, 3, 4, 5, 6, 7, 8, 9 and 10) and *Fuxianhuia xiaoshibaensis* (*n* = 4) (Additional file 1: Table S1). Note that the Y axis excludes the three reduced tergites that typify *Fuxianhuia* from total trunk tergite count

The available fossil material allows us to directly recognize 15 distinct ontogenetic stages based on the number and shape of the trunk tergites, and their allocation between the thorax and abdomen (Figs. 1, 3, 4, 5, 6, 7, 8, 9, 10 and 11). In addition to the three reduced anteriormost tergites - which are here functionally considered as part of the head region despite the lack of cephalic fusion - complete individuals possess between 8 and 30 trunk tergites according to their degree of ontogenetic development (Fig. 12a). Our staging scheme is based on the number of fully developed trunk tergites with lateral tergopleurae, excluding the three anteriormost reduced tergites because their precise segmental patterning remains uncertain until the discovery of even younger fossil individuals. In stage 8 – the youngest juveniles available in the existing fossil collections – the trunk consists of four limb-bearing thoracic tergites with short pleural spines (1:2 length/width ratio), and four limb-less abdominal tergites with a cylindrical outline (1:1 length/width ratio) (Figs. 1a, 3a*-*j). Thoracic tergites are approximately 1.5 times wider than those in the abdomen. Stage 9 is nearly identical to stage 8, differing only in the presence of five abdominal tergites (Fig. 1b; Fig. 3k*,* i). Individuals corresponding to stages 10 to 23 have not been recovered, but later ontogenetic phases demonstrate an increasing tergite count, and more substantial morphological differentiation. In stage 24, trunk tergites become broader and shorter, with the proportions being more pronounced in the tergites of the thorax (up to 1:6 length/width ratio) compared to those in the abdomen (1:2 length/width ratio) (Figs. 1c, 4a; Fig. 5), and thoracic tergites being twice as wide as those in the abdomen. This phase provides insight into the transition from abdominal into thoracic tergites during ontogeny. Stage 24 individuals display either 11 thoracic and 13 abdominal tergites (see stage 24a in Fig. 4; Fig. 5a*,* b), or 12 thoracic and 12 abdominal tergites (see stage 24b in Fig. 4; Fig. 5c*,* d). The transformation of the oldest abdominal tergite into the youngest thoracic tergite is demarcated by the appearance of short tergopleural spines in the latter, which subsequently expand laterally throughout ontogeny (Figs. 2a*,* 4a, 7c), as well as the appearance of walking legs, which increase in size and number of podomeres towards the anterior end (Fig. 2b) [1, 5]. Likewise, the trunk tergites in stage 25 are allocated as either 12 thoracic and 13 abdominal (see stage 25a; Fig. 6a*-*d), or 13 thoracic and 12 abdominal (see stage 25b; Fig. 6e*-*h). These observations reveal a biphasic developmental pattern in which the trunk alternates between events of *accumulation* where a new tergite is added from the posterior growth zone resulting in an abdomen with 13 tergites, followed by *depletion* where the most anterior abdominal tergite becomes morphologically differentiated and is incorporated into the thorax, leaving the abdomen with 12 tergites (Fig. 4). Critically, the thorax keeps incorporating new tergites with expanded tergopleurae one by one throughout ontogeny, increasing up to an observed maximum of 18 tergites (Figs. 1h*,* 4b; Figs. 10 and 11). This dynamic is maintained throughout later ontogeny (Fig. 1d*-*h; Figs. 6, 7, 8, 9 and 10), with only rare individuals demonstrating a slight deviation of the pattern, such as the early integration of a thoracic tergite that results in a shortened abdomen with only 11 tergites (Fig. 6i*,* j). Stage 30 is the most advanced developmental phase observed in our material, and is characterized by 18 thoracic and 12 abdominal tergites (Figs. 1h, 4a; Figs. 10 and 11). Here, tergite proportions are even more pronounced (thoracic, 1:13 length/width ratio; abdominal, 1:3 length/width ratio), and the thorax is up to four times wider than the abdomen. Taken together, the biphasic development and variability in the number of trunk tergites suggests that the complete ontogeny of *F. protensa* may have included up to 60 instar stages according to our staging scheme. This estimate carries the implication that the available material reflects approximately 25% of the post-embryonic development of this stem-group euarthropod, 20% of which is represented by advanced ontogenetic stages (Fig. 11).

Morphometric data obtained from previously published and our newly documented specimens of *F. protensa* indicate that overall body size (estimated from thoracic sagittal length) is positively correlated with the number of trunk tergites, and that there is a substantial degree of body size variation among later ontogenetic stages (Fig. 12b). This is partly owing to changes in the proportions of the dorsal exoskeleton throughout ontogeny, which complicate morphometric measurements of overall body size. For example, some of the stage 8 or 9 individuals have a similar thoracic length to that of stage 24 specimens (Fig. 12b). This can be explained by the difference in trunk tergite proportions between instars, as stage 8 and 9 individuals have proportionately elongate thoracic tergites, whereas the thoracic tergites of Stage 24 are relatively shorter and wider. The considerable overlap in size between different ontogenetic stages may also be a consequence of natural size variation between populations of specimens pooled together from several different localities within the Yu’anshan Member of the Chiungchussu Formation (Additional file 1: Table S1). Given that body size in extant euarthropods is a multifaceted phenomenon that is affected by both intrinsic and extrinsic factors [28], it is possible that environmental variables spanning short periods of geological time, such as local shifts in nutrient availability or temperature acting anywhere from annual seasons to centuries, could affect the rate of growth and maximum body size within the studied *F. protensa* populations. These differences could also be partly attributed to sexual dimorphism. However, discerning (at least some of) the primary effects responsible for controlling body size in *F. protensa* will only become possible through the additional input of completely articulated specimens corresponding to early juvenile stages.

**Fig. 13:**
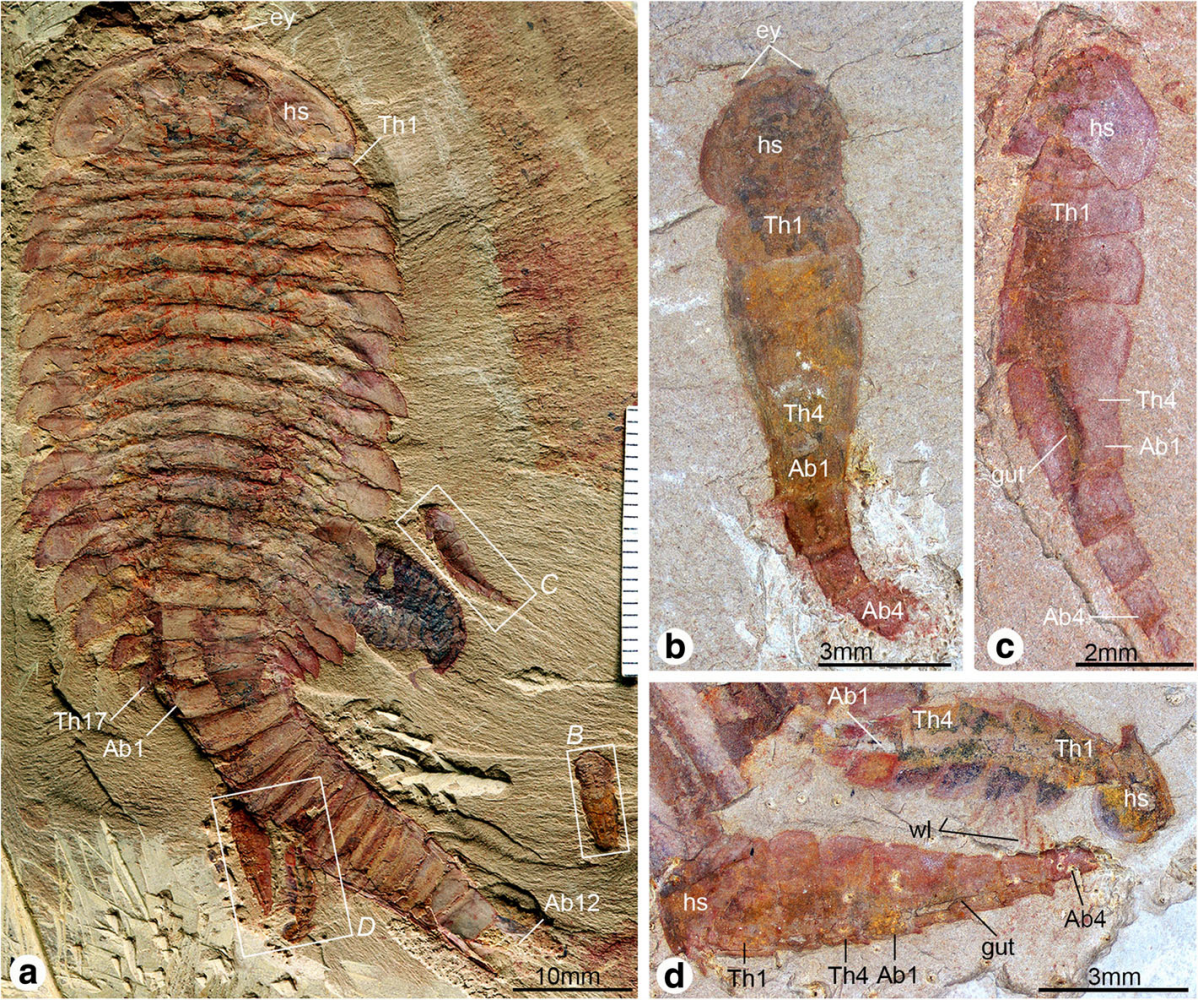
Fossil evidence for extended parental care in *Fuxianhuia protensa*. **a** ELI MU76A-a, life assemblage including a stage 29 – most likely sexually mature – adult individual and four stage 8 juveniles. **b** ELI MU76B-b, counterpart, articulated stage 8 juvenile with preserved eyes. **c** ELI MU76A-c, articulated stage 8 juvenile with preserved gut; note the presence of reduced tergites underneath head shield (see also Fig. 3). **d** ELI MU76A-d, two articulated stage 8 juveniles with preserved gut and walking legs. Abbreviations: Ab*n*, abdominal tergite; hs, head shield; tf, tail flukes; Th*n,* thoracic tergite; *wl*, walking legs

The new ontogenetic data on *F. protensa* allows the interpretation of a complex fossil specimen that consists of one stage 29 individual preserved in close association with four stage 8 juveniles (Fig. 9e*,* f; Fig. 13a-d). All five individuals are extremely well preserved, and display a substantial degree of integrity as observed from the completely articulated dorsal exoskeletons, as well as the presence of delicate structures such as the stalked eyes, head shield in life position, trunk appendages, and gut tract. These observations strongly suggest that the five individuals were preserved in situ, with only negligible transport or pre-burial disturbance, and thus in all likelihood reflect a life assemblage rather than a time-averaged aggregation of random carcasses or exuviae.

## Discussion

### The ontogenetic development of *Fuxianhuia protensa*

The new data on *F. protensa* represents the first detailed characterization of the post-embryonic development in an upper stem-group euarthropod [10, 12]. Despite the gap in the ontogenetic series between stage 9 and stage 24 (Fig. 11a; Fig. 12), the smaller individuals can be reliably identified as representatives of *F. protensa* based on their shared morphological features. The presence of stalked eyes connected to an anterior sclerite, ventral antennae, a pair of specialized post-antennal appendages, limb polypody, and a loosely attached head shield that covers multiple reduced anterior tergites are collectively strong indicators of fuxianhuiid affinities (Fig. 1a*,* b; Fig. 3) [1–5, 9, 11]. The dorsal exoskeleton of stages 8 and 9 resembles *Chengjiangocaris* [1, 2] and *Alacaris* [29] in the absence of a differentiated thoracic and abdominal regions; however, the possession of only three reduced anteriormost tergites underneath the head shield represents a key feature of *Fuxianhuia* [1, 2, 5, 11, 29], whereas most other genera of Fuxianhuiida possess five [1, 2, 29] or six [3] reduced tergites. Although the presence of three reduced tergites is also known in *Guangweicaris spinatus* from the Guanshan biota (Cambrian Stage 4, Wulongqing Formation) [4], this taxon has never been reported from any of the stratigraphically older Chengjiang localities despite substantial collecting efforts on the Chiungchussu Formation [1, 5, 6, 8, 11, 27, 30], and thus it is highly unlikely that the juvenile specimens are linked to it. In this context, the differences in the relative size and shape of the thoracic tergites between the *F. protensa* juveniles and adult specimens reflect ontogenetic change, as demonstrated by the gradual broadening of the thoracic tegites during stages 24 and 25 (Fig. 1a-d; Figs 3, 4, 5 and 6).

The ontogeny of *F. protensa* indicates that this taxon underwent anamorphic post-embryonic development (Figs. 4, 12, 13), in which new tergites were added sequentially from a posterior growth zone [19, 20, 24, 31, 32]. The biphasic developmental pattern of *F. protensa* is reminiscent of euarthropods that possess morphologically regionalized trunk regions, such as trilobites [24, 31, 32] and some crustaceans [21, 33, 34]. Trilobite development is broadly characterized as hemianamorphic; it consists of an anamorphic phase in which new segments are added from a posterior growth zone after each moult (accumulation), followed by an epimorphic phase with a fixed number of segments, which may involve their release from the fused pygidium into the freely articulating thorax (depletion), or a body size increase without significant morphological changes [24, 31, 32, 35]. Although the mode of ontogenetic growth is somewhat variable between different trilobite taxa [24], the biphasic growth of *F. protensa* bears some broad similarities with that of *Shumardia* (*Conophyrs*) *salopiensis* [24, 36]; both are typified by the alternation between posterior addition of segments, followed by the release of the anteriormost pygidial/abdominal segment into the thoracic region. Specifically, the pygidium of *S. salopiensis* alternates between three or four segments through part of its meraspid stage [24, 36], and thus superficially resembles the development of *F. protensa* in which the abdomen count varies between 12 and 13 tergites.

The growth trajectory of *F. protensa* as recorded by the morphometric data (Fig. 12b) also evokes parallels with that of the intensively studied Silurian proetid trilobite *Aulacopleura konincki*. Here, the range of body size is somewhat constrained among juvenile stages (meraspids), but becomes much more variable during late ontogeny resulting in mature adults (holaspids) with a polymorphic trunk segment count [37, 38]. The prevailing interpretation for the growth pattern of *A. konincki* favours a developmental model where the total number of thoracic segments was determined during early ontogeny, and once this maximum was reached, growth continued epimorphically in the holaspid stage. The small sample size of measurable juveniles available for *F. protensa* (stages 8 and 9, *n* = 9; Additional file 1: Table S1) prevents us from drawing comparable interpretations to those of *A. konincki* for the precise regulatory mechanisms responsible for trunk development (e.g. ontogenetically early versus late patterning, see ref. [37, 38]). However, the similar variability in body size relative to ontogenetic stage observed in late growth stages of *F. protensa* (Fig. 12b; trunk tergites versus thoracic length) and also holaspids of *A. konincki* (see fig. 3 in ref. [37]; thoracic tergites versus cranidial centroid size) suggests the existence of an epimorphic phase in the former fuxianhuiid. In this case, *F. protensa* growth would correspond to hemianamorphosis, as is also observed in numerous Cambrian euarthropods [12, 13, 19, 20, 24, 37, 38]. Although a definitive growth model is difficult to determine owing to the incompleteness of the early ontogenetic trajectory of *F. protensa*, the occurrence of hemianamorphic development in this taxon is in accord with most accepted interpretations for the ancestral mode of growth for crown-group Euarthropoda [19, 21, 24], as also suggested by the phylogenetic position of fuxianhuiids [9, 10, 12, 29]. Comparisons between the ontogenetic trajectories of *F. protensa* and *A. konincki* also raise the possibility that the former may have also exhibited adult polymorphism. Holaspids of *A. konincki* express five distinct morphotypes that possess between 18 and 22 freely articulating thoracic tergites, as well as a pygidium with three to seven segments [37, 38]. The five adult morphotypes of *A. konincki* correspond to the ontogenetic stages that show a substantial body size variation (estimated by cranidial centroid size) compared to the meraspids, namely those that have between 18 to 22 thoracic tergites (see fig. 3 in ref. [37]). When contrasted with the body size variation observed throughout development in *F. protensa* (Fig. 12b), the same parameter would suggest that this taxon may also possess multiple morphotypes, which would at least encompass stages 26 to 30. However, the full implications of this interpretation are yet again limited by the scarcity of juvenile *F. protensa* material that would inform on the entire growth trajectory, and the mixed provenance of the fossils from several localities (Additional file 1: Table S1).

Among extant euarthropods, the biphasic development of the dorsal exoskeleton in *F. protensa* evokes comparison with that of Cephalocarida, a relatively poorly understood but highly ecologically specialized clade of meiofaunal pancrustaceans [33, 34, 39]. The post-cephalic exoskeleton of cephalocarids comprises a limb-bearing thorax with pivot-jointed tergites that possess laterally extended tergopleurae, followed by an elongate and flexible abdomen formed by cylindrical tergites without appendages. During their early anamorphic development, the trunk of cephalocarids alternates between the production of new abdominal segments from a posterior growth-zone, and the transformation of the anteriormost abdominal tergite into a limb-bearing thoracic tergite with expanded tergopleurae [33, 34]. Although there is some variation on the timing of these events between cephalocarid species [33], this biphasic pattern resembles the ontogenetic development of the trunk in *F. protensa* (Figs. 4, 12, 13). The peculiar development of Cephalocarida has been considered as autapomorphic among crustaceans [33], which suggests that the growth mode of *F. protensa* most likely evolved independently given the phylogenetic position of Fuxianhuiida within the euarthropod stem lineage [10–12, 29]. This interpretation is further supported by the lack of substantial dorsal exoskeletal tagmosis in closely related taxa such as *Chengjiangocaris* [1, 5]*, Liangwangshania* [3, 40] and *Alacaris* [29]. Ultimately, the ontogeny and phylogenetic position of *F. protensa* support anamorphosis – and particularly hemianamorphosis – as the ancestral mode of euarthropod post-embryonic development [12, 13, 18–21, 24], whereas the dorsal exoskeletal tagmosis of *Fuxianhuia* species [1, 2, 5] - and the closely related *Guangweicaris* [4] - are best regarded as a derived condition within upper stem-group Euarthropoda [10–12, 29].

### Trunk segmentation

Our data also offer additional insights into the segmental organization of fuxianhuiids. It is well established that the thoracic tergites of *F. protensa* bear more than one set of biramous limbs, which raises the question of whether this type of segmental mismatch results from the derived organization of the dorsal or ventral side of the trunk [1, 2, 5, 9, 41]. The formation of the tergites from the posterior growth zone in the abdomen (Figs. 4, 11, 12), together with their release into the thoracic region and subsequent lateral expansion of the tergopleurae, suggest that the dorsal exoskeleton of fuxianhuiids follows the conventional pattern of segment production observed in most euarthropods [19–22, 31, 32, 35]. This condition implies that the presence of multiple leg pairs associated with each thoracic tergite most likely represents a derived mode of ventral segmentation exclusive to this body region, further supported by the recent discovery of metamerically arranged midgut diverticulae in *F. protensa* that match the dorsal segmentation pattern [27]. Although rare, a similar type of dorsoventral segmental mismatch is also observed in the ventral trunk of the branchiopod *Triops cancriformis* [42, 43]. The anamorphic growth of *T. cancriformis* comprises the formation of limb-less abdominal segments from a posterior growth zone, which then develop between three and four pairs of limb buds that result in supernumerary thoracopods per segment in the adult [42]. This condition is closely reminiscent to that of *F. protensa*, including the fact that the newly formed posterior thoracic appendages are less developed compared to those on the anterior end of the body (Fig. 2).

### Pisinnocaris as junior synonym of Fuxianhuia

The new data on the ontogenetic development of *F. protensa* has direct implications for the diversity of fuxianhuiids from Chengjiang-type biotas in South China. Juveniles corresponding to stages 8 and 9 (Fig. 1a,b; Fig. 3) closely resemble the enigmatic euarthropod *Pisinnocaris subconigera* [43]. *Pisinnocaris* is only known from few specimens that reach a maximum length of 10.7 mm, and thus falls within the size range of stage 8 juveniles of *F. protensa* (Figs. 3, 4, 11, 12). The morphology of *Pisinnocaris* consists of a pair of stalked eyes followed by uniramous antennae, a subtrapezoidal head shield, and a trunk composed of at least 8 or 9 tergites that tapper in width posteriorly. The type material does not preserve walking legs, but shows traces of possible attachment sites that suggest the presence of several limb pairs per tergite. It is possible to subdivide the trunk into a thoracic region based on the presence of short tergopleural spines, whereas these are absent on the comparatively more narrow abdominal tergites [43]. Although the available material of *Pisinnocaris* from the Chengjiang does not demonstrate the presence of reduced anterior tergites underneath the head shield as observed in juveniles of *F. protensa* (Fig. 3), all other aspects of the exoskeletal organization are identical between these taxa. Furthermore, Zeng et al. [44] recently illustrated a single specimen of a small (ca. 6 mm long) euarthropod tentatively assigned to *Pisinnocaris* based on the morphology of the head shield and anterior trunk region from the Cambrian (Stage 3) Hongjinshao Formation at Chenggong. This specimen is highly significant, as it unequivocally demonstrates the presence of reduced anterior tergites underneath the head shield, and traces of multiple limbs associated with the thoracic tergites. Although the posterior half of the Chenggong euarthropod is not preserved, its morphology and size fit comfortably within those of stage 8 or 9 juveniles of *F. protensa* (Figs. 3, 4, 11, 12). Collectively, these observations indicate that *Pisinnocaris* actually corresponds to a juvenile of *Fuxianhuia*, rather than a separate taxon; thus, *Pisinnocaris* is best regarded as a junior synonym of *Fuxianhuia.*

Xu [45] described a single Chengjiang euarthropod specimen as *Pisinnocaris* sp. based on the morphology of the head shield and similar trunk organization. Although *Pisinnocaris* sp. could be of potential relevance to the present study given the possession of 11 trunk tergites, the single specimen does not demonstrate the presence of reduced tergites, nor the morphological differentiation of the trunk into a thoracic and abdominal region. The trunk tappers in width posteriorly, but clearly features well-developed posterior-facing tergopleural spines throughout the entire length of the body (see Plate 1, figure 3 in [45]). *Pisinnocaris* sp. also differs from *F. protensa* early juveniles in having an elongate and robust tailspine with a subrectangular outline, and no evidence of the commonly preserved tail flukes (Figs. 3, 4, 5, 6, 7, 8, 9 and 10). More critically, the only known specimen of *Pisinnocaris* sp. reaches a body length of 47 mm, making it more than three times larger than known juveniles of *F. protensa* (ca. 15 mm max. length). In this context, the overall exoskeletal dorsal morphology and size of *Pisinnocaris* sp. most closely resemble those of the aglaspidid euarthropod *Tremaglaspis* [46–48], potentially extending the stratigraphic range of this taxon to the Cambrian Stage 3. Given the rarity of *Pisinnocaris* sp. within the Chengjiang, we refrain from formalizing this interpretation until more specimens of this problematic taxon become available for study and comparison with the type material of *Tremaglaspis*.

### Palaeoecological implications

The post-embryonic ontogeny of *F. protensa* is characterized by the addition of tergites from a posterior growth-zone, as well as the corresponding increase in size (Figs. 4, 11, 12). Comparisons between body size increments and the number of trunk tergites reveal a substantial degree of variability in *F. protensa*, and indicate that there is not a simple or direct correlation between these parameters during ontogeny (Fig. 12b). This pattern resembles the flexible thoracic segmentation observed in some Cambrian olenimorphic trilobites – which are characterized by having narrow axis, extended terpleurae and segment-rich thorax – as well as the Silurian trilobite *A. konincki* [37, 38, 49–51]. The flexibility in adult segment number observed in olenimorphic trilobites suggests that the variable trunk segmentation of *F. protensa* could potentially represent an adaptation for environmental conditions with reduced oxygen, in which the presence of numerous pairs of walking legs – and their corresponding flap-like exopods – would maximize ventilation for respiration [51, 52]. Geochemical data [53] and the scarcity of trace fossils in these deposits [30], which indicate that the Chengjiang environment would have experienced fluctuating oxygen levels, offers some support to the adaptive value of flexible patterns of trunk segmentation and polypody in *F. protensa* by analogy with Cambrian and Silurian olenimorphic trilobites [37, 38, 51].

### Morphological diversification and heterochrony in fuxianhuiid evolution

The growth pattern of *F. protensa* allows further comparisons with the congeneric – and stratigraphically younger – *F. xiaoshibaensis* [2, 29, 44]. Despite their close morphological similarities, *F. xiaoshibaensis* differs from *F. protensa* in the higher trunk tergite count, more specifically in the presence of up to 17 thoracic and 16 abdominal tergites. Although the higher tergite count of *F. xiaoshibaensis* could be explained as a result of continued development in *F. protensa*, the ontogenetic trajectories of these taxa argue against this interpretation (Fig. 12b). Individuals of *F. xiaoshibaensis* are up to three times smaller compared to advanced ontogenetic stages of *F. protensa*; these differences suggest an accelerated rate of tergite addition during early ontogeny in *F. xiaoshibaensis* relative to the stratigraphically older *F. protensa*, and fall in accordance to peramorphic heterochronic development [54–56]. This phenomenon is clearly expressed in the presence of 16 abdominal tergites of *F. xiaoshibaensis,* compared to the 12 – or exceptionally 13 – abdominal tergites of *F. protensa*, despite both species having a comparable maximum number of thoracic tergites*.* However, the restricted number of completely articulated specimens of *F. xiaoshibaensis* precludes further investigations on this matter (Fig. 12b), and thus this hypothesis awaits corroboration through the analysis of additional material.

### Extended parental care in *Fuxianhuia*

**Fig. 14:**
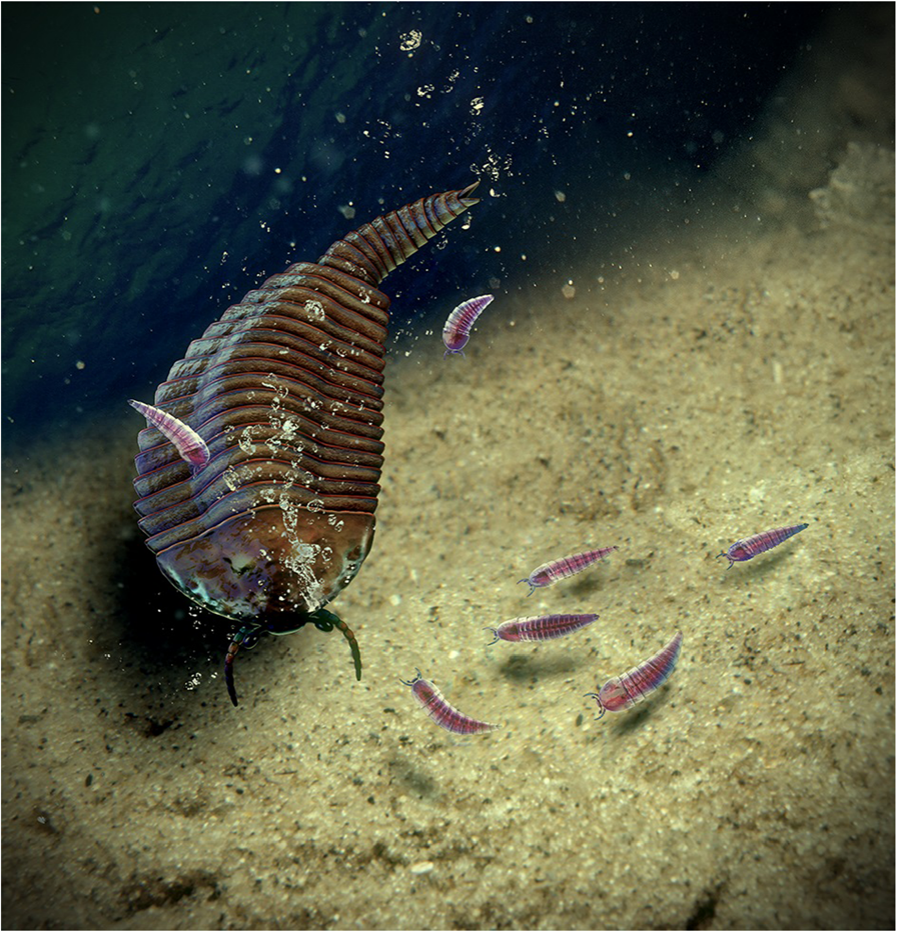
Reconstruction of extended parental care in *Fuxianhuia protensa*

Given the data available on the post-embryonic ontogenetic development of *F. protensa* and the improved understanding of its morphological variability, we interpret the exceptionally preserved in situ association of a stage 29 individual alongside four stage 8 juveniles as evidence of a parent and its offspring respectively (Figs. 9e-f, 13a, 14). Although it is not possible to pinpoint the exact growth stage at which fuxianhuiids became able to reproduce, the ontogeny of *F. protensa* identifies the stage 29 individual as developmentally advanced (Figs. 4, 11, 12), and thus in all likelihood a sexually mature adult. The fact that all the juveniles correspond to stage 8, and therefore have implicitly undergone a degree of post-embryonic development, identifies this association as a case of extended parental care, in which the parent nurtures the offspring during early ontogeny to maximize their survival and fitness [57–59]. This interpretation is supported by the fact that the four juveniles are ontogenetically contemporaneous, suggesting that they all belong to the same clutch. Unlike some cases of extended parental care in extant euarthropods or stratigraphically younger fossils, the adult *F. protensa* has no obvious morphological adaptations for nursing the juveniles. For example, peracarid adult females have a specialized pouch for carrying the eggs and early juveniles [60, 61], and in some decapods the offspring may attach directly to the body of the parent [57, 58]. However, there are also recorded instances of extended parental care in which the parent cohabits with the juvenile for a prolonged period after hatching without the aid of specialized structures [62]. In the absence of a brood pouch or other similar feature in *F. protensa*, we contend that this exceptional fossil association captures a case of prolonged cohabitation between parent and offspring (Figs. 9e*,* f; Figs. 13, 14), not dissimilar from that observed in some extant marine crustaceans [57–59, 62]. Thus, *F. protensa* provides the phylogenetically and stratigraphically earliest evidence of extended parental care in the euarthropod fossil record [15–18, 60, 63], and reveals a greater diversity and complexity of reproductive strategies during the early Cambrian well beyond that of egg brood-care within a bivalved carapace [15–18].

## Conclusions

Our survey of completely articulated specimens of *F. protensa* from the early Cambrian Chengjiang cast new light on several aspects of the post-embryonic ontogenetic development, palaeobiology, and reproductive behaviour of these important upper stem-group euarthropods. The recognition of anamorphosis – and likely hemianamorphosis – in *F. protensa* provide a minimum constrain for the origin of this ancestral growth mode for the euarthropod stem lineage. However, these findings also demonstrate that phylogenetically early diverging euarthropods already possessed a substantial degree of developmental variability, expressed in *F. protensa* in the biphasic pattern of tergite addition from a posterior growth zone, and the available evidence suggesting polymorphism in the number of adult thoracic tergites. Although there is still much to learn about the development of stem group euarthropods, the new data on *F. protensa* fills a critical gap in our understanding of the evolution of post-embryonic ontogeny, and highlights the significance of development as a tool for a more holistic understanding of the palaeobiology of extinct organisms.

## Methods

The studied fossils are the result of decades of collecting effort from various localities of the Cambrian (Stage 3; locally Qiongzhusian) Yu’anshan Member of the Chiungchussu Formation of the Kunming region in South China [30]. The material is deposited in the Early Life Institute (ELI), Northwest University, Xi’an (see Additional file 1: Table S1). Specimens were prepared with fine needles under high magnification using stereomicroscopes. Fossils were photographed with a Canon EOS 5D Mark II digital camera and were processed in Adobe Photoshop CS 5. Camera lucida and interpretative drawings were made using a Leica M80 microscope and prepared with Corel Draw X5. Morphometric data were measured from specimen digital photographs using the software FIJI [64].

## Additional file

Additional file 1: **Table S1.** Morphometric and locality data of articulated individuals of *Fuxianhuia*. (XLSX 15 kb)
